# Alcohol intake, tobacco smoking, and esophageal adenocarcinoma survival: a molecular pathology epidemiology cohort study

**DOI:** 10.1007/s10552-019-01247-2

**Published:** 2019-11-30

**Authors:** R. Stephen McCain, Damian T. McManus, Stephen McQuaid, Jacqueline A. James, Manuel Salto-Tellez, Nathan B. Reid, Stephanie Craig, Chintapuza Chisambo, Victoria Bingham, Eamon McCarron, Eileen Parkes, Richard C. Turkington, Helen G. Coleman

**Affiliations:** 1 Cancer Epidemiology Research Group, Centre for Public Health, Queen’s University Belfast, ICS-B Building, RVH Site, Grosvenor Road, Belfast, BT12 6BJ Northern Ireland; 2 Department of Pathology, Belfast Health and Social Care Trust, Belfast, Northern Ireland; 3 Centre for Cancer Research and Cell Biology, Queen’s University Belfast, Belfast, Northern Ireland

**Keywords:** Epidemiology, Esophageal cancer, Lifestyle, Biomarkers, Prognosis

## Abstract

**Purpose:**

To investigate the association between cigarette smoking, alcohol consumption, and esophageal adenocarcinoma survival, including stratified analysis by selected prognostic biomarkers.

**Methods:**

A population-representative sample of 130 esophageal adenocarcinoma patients (n = 130) treated at the Northern Ireland Cancer Centre between 2004 and 2012. Cox proportional hazards models were applied to evaluate associations between smoking status, alcohol intake, and survival. Secondary analyses investigated these associations across categories of p53, HER2, CD8, and GLUT-1 biomarker expression.

**Results:**

In esophageal adenocarcinoma patients, there was a significantly increased risk of cancer-specific mortality in ever, compared to never, alcohol drinkers in unadjusted (HR 1.96 95% CI 1.13–3.38) but not adjusted (HR 1.70 95% CI 0.95–3.04) analysis. This increased risk of death observed for alcohol consumers was more evident in patients with normal p53 expression, GLUT-1 positive or CD-8 positive tumors. There were no significant associations between survival and smoking status in esophageal adenocarcinoma patients.

**Conclusions:**

In esophageal adenocarcinoma patients, cigarette smoking or alcohol consumption was not associated with a significant difference in survival in comparison with never smokers and never drinkers in fully adjusted analysis. However, in some biomarker-selected subgroups, ever-alcohol consumption was associated with a worsened survival in comparison with never drinkers. Larger studies are needed to investigate these findings, as these lifestyle habits may not only be linked to cancer risk but also cancer survival.

## Introduction

Esophageal cancer has a rising incidence and is the eighth most common cancer worldwide with 572,034 new diagnosis made in 2018 [1]. Esophageal squamous cell carcinoma continues to be the predominant esophageal cancer; however, in the last three to four decades, there has been a dramatic shift in the demographics of the histological sub-type of esophageal cancer diagnosed. In the developed world, there is a decreased incidence of squamous cell carcinoma, and a simultaneous increase in the incidence of adenocarcinoma [1, 2]. For example, in the USA, between 1975 and 2004, there was a 463% increase in esophageal adenocarcinoma and similar patterns have been seen in the UK, and Western Europe where the incidence of esophageal adenocarcinoma now outnumbers the incidence of esophageal squamous cell carcinoma [1, 3]. Esophageal adenocarcinoma 5-year survival rates in the Western world range from 10 to 18% [4, 5] and this is partially due to only 38% of patients being suitable to undergo treatment with a curative intent [6]. However, even within patients with localized disease that undergo attempted curative resection, the 5-year survival is still as low as 47% which highlights the need for additional research in an effort to improve survival rates [7].

The impact of lifestyle factors on esophageal adenocarcinoma development has been investigated extensively. For example, the BEACON consortium demonstrated a strong association between cigarette smoking and esophageal adenocarcinoma development [8], and therefore, it could be hypothesized that cigarette smoking may also impact upon survival in esophageal adenocarcinoma. Although there has been shown to be no association between alcohol consumption and esophageal adenocarcinoma risk [9], it is worthwhile investigating if alcohol consumption plays a role in prognosis as it is an easily modifiable risk factor and is known to have a synergistic effect with cigarette smoking in other cancers. However, only four published studies, including relatively small numbers of patients, have investigated the association between tobacco smoking, alcohol consumption, and esophageal adenocarcinoma survival [10–13]. Our working group has published a recent meta-analysis combining two of these studies, which demonstrated no significant difference in esophageal adenocarcinoma survival in never-alcohol drinkers compared to moderate alcohol drinkers (HR 1.34 95% CI 0.95–1.89) [14]. Similarly, there were no associations with survival in esophageal adenocarcinoma patients who were current (HR 0.99 95% CI 0.73–1.36) or former (HR 0.88 95% CI 0.68–1.14) smokers, compared to never smokers [14]. This meta-analysis also investigated the association between these lifestyle factors and survival in other cancers of the digestive tract. Results demonstrated that cigarette smoking was associated with poorer survival in patients with colorectal, gastric, or pancreatic cancer, hepatocellular carcinoma, or esophageal squamous cell carcinoma [14]. Alcohol consumption was associated with a poorer survival in patients with esophageal squamous cell carcinoma or hepatocellular carcinoma [14]. Given the current dearth of research on the association between these lifestyle factors and esophageal adenocarcinoma survival, further studies are necessary.

The primary aim of this study is to investigate the potential association between cigarette smoking, alcohol consumption, and esophageal adenocarcinoma survival. A secondary aim was to investigate the impact of these lifestyle factors on survival, according to the expression of the selected biomarkers, P53, HER2, GLUT1, and CD8 which have been shown to be associated with prognosis in esophageal adenocarcinoma [15–18].

A higher expression of P53, HER2, and GLUT1 has individually been shown to be associated with a worse survival in esophageal adenocarcinoma [19]. However, there have been a variety of other cancers which have been investigated with a similar methodology. In esophageal squamous cell carcinoma, heavy smokers have been shown to have a two-times higher odds of P53 mutation than non-smokers [20], and in lung cancer, frequent alcohol drinkers had a 4.6-fold increased odds of having a P53 mutation [21]. Several studies in breast cancer have not shown any association between alcohol consumption and HER2 receptor expression [22] and to date, no studies have reported on the impact of cigarette smoking or alcohol consumption on GLUT1 expression in any type of cancer. A higher CD8+ tumor expression in esophageal adenocarcinoma has been shown to be associated with a significantly improved overall survival [23]. Despite these findings, no previous studies have investigated the impact of smoking or alcohol consumption on esophageal adenocarcinoma survival according to strata of these biomarkers, which is an important consideration for future precision medicine initiatives. This study represents a molecular pathology epidemiology approach, which has not been extensively applied in esophageal cancer survival studies to date [24].

## Methods

This study was performed and reported in line with the REMARK guidelines [25].

### Patient selection

**Fig. 1:**
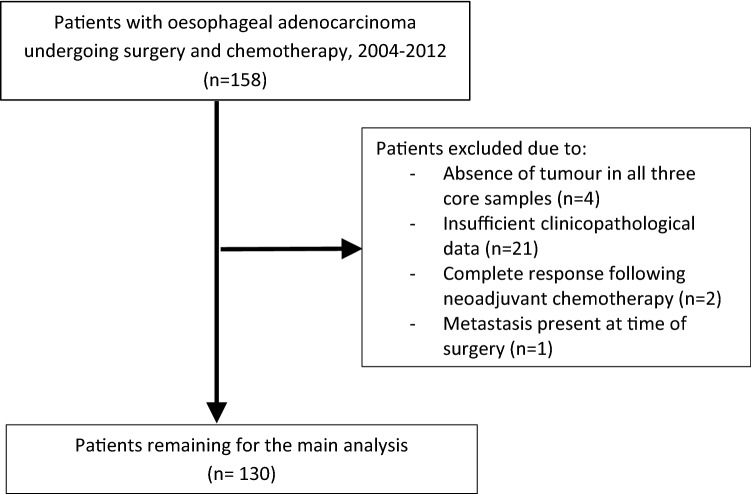
Flow chart demonstrating reasons for patient exclusion from study

In this population-representative study, all patients in Northern Ireland who underwent neoadjuvant chemotherapy followed by surgical resection for esophageal adenocarcinoma between 1 January 2004 and 31 December 2012 were identified. There were 158 corresponding formalin-fixed paraffin embedded (FFPE) esophageal adenocarcinoma resection specimens collected from the Northern Ireland Cancer Centre. Of these, matched clinical information was available for 137 patients, but seven patients (for the reasons outlined in Fig. 1) were excluded, leaving 130 patients for inclusion in the primary analysis. Relevant ethical approvals were obtained from the Northern Ireland Biobank (NIB12-0032 and NIB12-0062) and the Office for Research Ethics Committees Northern Ireland (ORECNI, 13/NI/0149) [26]. The staining and study of the biomarker CD8 was performed under the accelerator grant from Cancer Research UK (C11512/A20256 to PWH/MS-T).

### Clinical data

Clinical data and information on study outcomes up until 31 December 2014 was retrieved via patient note review at the Northern Ireland Cancer Centre, as previously described [17]. Patient information included age at diagnosis, date of diagnosis, date of surgery, and patient sex. Tobacco smoking status was classified as never, current, and former smokers, as recorded within medical notes. Alcohol consumption was classified as never and ever drinkers, as recorded in patient notes. Those patients with an unknown smoking and alcohol status were also analyzed separately to evaluate if they had differential associations between exposures and survival outcomes.

Pathology reports from resection specimens were reviewed for tumor characteristics including tumor location, presence of lymphovascular invasion, circumferential resection margin status, tumor differentiation, and TNM stage. Tumor location was divided into lower third of esophagus (greater than 5 cm proximal to the esophagogastric junction), Siewert 1 (within 1–5 cm above the oesophagogastric junction), Siewert 2 (within 1 cm above and 2 cm below the oesophagogastric junction), and Siewert 3 (2–5 cm below the oesophagogastric junction) [27]. Pathological staging was defined according to International Union Against Cancer (UICC) TNM staging, 7th edition [28]. Finally, the date and cause of death were recorded, where applicable. The survival time was calculated from the day of diagnosis to the date of death.

### Construction of tissue microarrays

A FFPE tissue block was selected from each resection specimen and triplicate 1 mm cores of tumor were embedded in a paraffin block using the Beecher Manual Arrayer® to enable tumor samples to be stained and scored.

### Immunohistochemistry staining and scoring

Immunohistochemical analysis was performed within the Northern Ireland Molecular Pathology Laboratory at Queen’s University Belfast, following approval by the Northern Ireland Biobank. To enable biomarker expression to be evaluated, slides were scanned on an Aperio AT2 scanner, and viewed as digital images on Xplore (PathXL) and then manually scored. The validated antibodies and techniques used for immunohistochemical staining are presented in Table 1. Detailed scoring methods are described below for the biomarkers investigated (HER2, p53, GLUT1, and CD8), which were all chosen due to previous publications demonstrating their prognostic ability and/or prevalent staining in esophageal adenocarcinoma tumors [15–18]. Their interaction with lifestyle factors to influence prognosis is not yet known, and this analysis should be regarded as hypothesis-generating.

**Table 1:** Antibodies and techniques used for each method of immunohistochemical staining

Antibody	Clone	Supplier	Catalog number	Species	Platform	Retrieval	Antibody dilution and incubation	Detection chemistry
p53	DO-7	Dako	M7001	Mouse	Bond RX	Epitope retrieval solution 1 (ER1) 30MINS	1/1000 15MINS	Bond polymer DAB refine + Enhancer
GLUT-1	NA	Roche Ventana	760-4526	Rabbit polyclonal	Ventana benchmark	Cell conditioning solution 1 (CC1) 8 min	pre diluted	Optiview DAB Kit
CD8	C8/144B	Dako	M7103	Mouse	Bond RX	Epitope retrieval solution 2 (ER2) 20mins	1/50 15mins	Bond polymer DAB refine + enhancer
HER4B5	4B5	Roche Ventana	790-2991	Rabbit	Ventana benchmark	Cell conditioning solution 1 (CC1) 30 min	pre diluted	Ultraview DAB IHC Kit

PET responder defined as a reduction in standardized uptake values of    > 35%

Positive circumferential resection margin defined as tumor at or within 1 mm of the circumferential resection margin

*PET*-positive emission tomography scan, *T stage* tumor stage, *N stage* nodal stage

## p53

The p53 staining was performed as previously described [29]. The nuclear staining intensity and percentage of the tumor cell’s nucleus staining positive in sections of TMA cores were assessed by two independent observers and a final agreement on discordant results was made. Scoring was based on intensity (0 = no staining, 1 = weak, 2 = moderate, and 3 = strong staining observed) and the percentage of tumor cells staining positive (0–100%). These two scores were multiplied to give an H-score between 0 and 300. Triplicate scores were taken for each patient and the maximum score was used for statistical analysis. Patients were then divided into tertiles of p53 expression with the cutoffs for the tertiles being < 80, 80 to  < 240, and > 240 which was based on the distribution of p53 expression scores for the included patients. For this study, we describe the middle tertile of 80 to < 240 as the normal range of p53 expression.

## GLUT1

GLUT1 has been identified as a marker of poor prognosis in esophageal adenocarcinoma but its interaction with lifestyle factors is not known [17]. Staining was scored by three independent observers as previously described [17], with agreement made on discordant results. If any cancer cell membrane or cytoplasm stained positive for GLUT1. the tumor was considered to be GLUT1-positive and patients were enabled division into groups of GLUT1-positive and GLUT1-negative tumors.

## HER-2

HER2 expression was evaluated by assessing the degree of expression in the tumor cell population. Staining was scored by three observers in keeping with the accepted method in the UK and USA as follows: HER2 0− negative no staining or membrane staining is < 10% of cancer cells, 1+ negative faint/barely perceptive membrane staining in more than 10% of the cancer cells, 2+ equivocal weak to moderate complete membrane staining in more than 10% of cancer cells or < 30% with strong complete membrane staining, and 3+ is considered positive and involves strong complete membrane staining in more than 30% of cancer cells [30]. Following application of these scoring methods, patients were divided into HER-2-positive and HER-2-negative tumor categories.

## CD8

Scoring for CD8 was performed by two independent observers with a final agreement made on discordant results. A semiquantitative scoring system was employed for CD8 characterization based on the intensity of staining within intratumoural tissue. A score of 3 indicates strong CD8 expression, 2 moderate expression, 1 low or weak expression, and 0 absence. Patients were divided into groups of either CD8-positive (1, 2, 3) or CD8-negative tumors.

### Statistical analysis

Patient demographics and tumor characteristics according to smoking status and alcohol consumption were compared using chi-squared tests.

Overall survival (death from any cause) and cancer-specific survival (death from oesophageal adenocarcinoma) were evaluated using Cox proportional hazards regression models for unadjusted and adjusted results. The variables included in the adjusted analysis were age at diagnosis, gender, tumor nodal status, circumferential resection margin, tumor differentiation, lymphovascular invasion, and tumor location. In further analyses, alcohol consumption and smoking status were mutually adjusted for each other. Adjustments for tumor T stage did not influence the model, and therefore, it was omitted from final survival analysis.

Hypothesis-generating survival analysis was performed for smoking and alcohol status stratified by categories of tumor biomarker expression. There were different numbers of patients within each biomarker study as not all cores taken from the TMA from each biomarker staining had the presence of tumor . There were 130 TMA cores included for p53, 130 for HER-2, 129 for GLUT-1, and 100 for CD8. In this analysis, patients were divided into never and ever smokers compared to the never, current, and former used in the primary analysis as the latter, due to relatively small sample sizes in these strata. Stata version 14.2 (College Station, TX, USA) was used for statistical analysis.

## Results

### Patient demographics and tumor characteristics

Of the total 130 oesophageal adenocarcinoma patients in this study, 78% were male and 22% were female and the majority of patients were over 60 years old (70%). The majority of tumors were located at the gastro-esophageal junction (84.6%), with Siewert 1 tumors the most common (50.8%), followed by Siewert 2 (25.4%) and Siewert 3 (8.5%).

Table 2 presents the patient demographics and tumor characteristics across categories of smoking and alcohol status. There was no difference by patient sex, tumor site, lymphovascular invasion status, circumferential resection margin status, tumor differentiation, tumor T stage, or surgical nodal status according to categories of smoking and alcohol status. Current and former smokers were more likely to be older than non-smokers (*p* = 0.02).

**Table 2:** Patient demographics and tumor characteristics according to smoking and alcohol status

	Total *n* = 130	Smoking Status					Alcohol status			
		Never	Current	Former	Unknown	*p*-value	Never	Ever	Unknown	*p*-value
Sex										0.20
Male	101 (77.7)	24 (70.6)	22 (71)	41 (85.4)	14 (82.4)	0.30	28 (68.3)	56 (81.2)	17 (85)	
Female	29 (22.3)	10 (29.4)	9 (29)	7 (14.6)	3 (17.6)		13 (31.7)	13 (18.8)	3 (15)	
Age at diagnosis (years)										
< 50	14 (10.8)	5 (14.7)	6 (19.4)	3 (6.3)	0 (0)	0.02	4 (9.8)	10 (14.5)	0 (0)	0.25
50–59	25 (19.2)	5 (14.7)	11 (35.5)	8 (16.7)	1 (5.9)		6 (14.6)	16 (23.2)	3 (15)	
60–69	61 (46.9)	16 (47.1)	13 (41.9)	23 (47.9)	9 (52.9)		22 (53.7)	30 (43.5)	9 (45)	
≥ 70	30 (23.1)	8 (23.5)	1 (3.2)	14 (29.2)	7 (41.2)		9 (22)	13 (18.8)	8 (40)	
Primary site										
Lower third	20 (15.4)	2 (5.9)	6 (19.4)	9 (18.8)	3 (17.7)	0.36	8 (19.5)	7 (10.1)	5 (25)	0.18
Gastro-esophageal junction	110 (84.6)	32 (94.1)	25 (80.6)	39 (81.3)	14 (82.3)		33 (80.5)	62 (89.9)	15 (75)	
Siewert classification										
1	66 (50.8)	18 (52.9)	12 (38.7)	28 (58.3)	8 (47.1)	0.26	19 (46.3)	38 (55.1)	9 (45)	0.67
2	33 (25.4)	8 (23.5)	11 (35.5)	9 (18.7)	5 (29.4)		11 (26.8)	17 (24.6)	5 (25)	
3	11 (8.5)	6 (17.7)	2 (6.4)	2 (4.2)	1 (5.9)		3 (7.3)	7 (10.1)	1 (5)	
PET responder										
No	43 (33.1)	14 (41.2)	12 (38.7)	14 (29.2)	3 (17.7)		15 (36.6)	25 (36.2)	3 (15)	0.10
Yes	57 (43.9)	13 (38.2)	13 (41.9)	23 (47.9)	8 (47.1)	0.64	21 (51.2)	27 (39.1)	9 (45)	
Unknown	30 (23.1)	7 (20.6)	6 (19.4)	11 (22.9)	6 (35.3)		5 (12.2)	17 (24.6)	8 (40)	
Lymphatic vascular invasion										
Yes	90 (69.2)	25 (73.5)	20 (64.5)	32 (66.7)	13 (76.5)	0.62	24 (58.5)	52 (75.4)	14 (70)	0.29
No	39 (30)	8 (23.5)	11 (35.5)	16 (33.3)	4 (3.5)		16 (39)	17 (24.6)	6 (30)	
Unknown	1 (0.8)	1 (3)	0 (0)	0 (0)	0 (0)		1 (2.5)	0 (0)	0 (0)	
Circumferential resection margin										
Negative	72 (55.4)	20 (58.8)	16 (51.6)	25 (52.1)	11 (64.7)	0.84	24 (58.5)	35 (50.7)	13 (65)	0.70
Positive	57 (43.8)	14 (41.2)	15 (48.4)	22 (45.8)	6 (35.3)		17 (41.5)	33 (47.8)	7 (35)	
Unknown	1 (0.8)	0 (0)	0 (0)	1 (2.1)	0 (0)		0 (0)	1 (1.5)	0 (0)	
Differentiation										
Well	2 (1.5)	1 (2.9)	1 (3.23)	0 (0)	0 (0)	0.39	0 (0)	2 (2.9)	0 (0)	0.59
Moderate	50 (38.5)	16 (47.1)	14 (45.2)	16 (33.3)	4 (23.5)		17 (41.5)	26 (37.7)	7 (35)	
Moderate–poor	14 (10.8)	4 (11.8)	2 (6.5)	4 (8.3)	4 (23.5)		5 (12.2)	5 (7.3)	4 (20)	
Poor	64 (49.2)	13 (38.2)	14 (45.2)	28 (58.3)	9 (52.9)		19 (46.3)	36 (52.3)	9 (45)	
Surgical T stage										
ypT1	11 (8.5)	3 (8.8)	2 (6.5)	5 (10.4)	1 (5.9)	0.93	3 (7.3)	7 (10.1)	1 (5)	0.55
ypT2	25 (19.2)	5 (14.7)	7 (22.6)	8 (16.7)	5 (29.4)		11 (26.8)	9 (13)	5 (25)	
ypT3	89 (68.5)	25 (73.5)	21 (67.7)	32 (66.7)	11 (64.7)		25 (61)	50 (72.5)	14 (70)	
ypT4	5 (3.9)	1 (2.9)	1 (3.2)	3 (6.3)	0 (0)		2 (4.9)	3 (4.4)	0 (0)	
Surgical N stage										
ypN 0	44 (33.9)	9 (26.5)	9 (29)	19 (39.6)	7 (41.2)	0.60	16 (39)	18 (26.1)	10 (50)	0.16
ypN1	27 (20.8)	6 (17.7)	7 (22.6)	11 (22.9)	3 (17.7)		9 (22)	15 (21.7)	3 (15)	
ypN2	29 (2.3)	11 (32.4)	8 (25.8)	9 (18.8)	1 (5.9)		7 (17)	21 (30.4)	1 (5)	
ypN3	30 (23.1)	8 (23.5)	7 (22.6)	9 (18.8)	6 (35.3)		9 (22)	15 (21.7)	5 (6)	

PET responder defined as a reduction in standardized uptake values of > 35%

Positive circumferential resection margin defined as tumor at or within 1 mm of the circumferential resection margin

*PET*-positive emission tomography scan, *T stage* tumor stage, *N stage* nodal stage

### Survival analysis

There were 75 patients who died during a maximum of 9 (median 2.5) years of follow-up, with 70 of these patients having a recorded cause of death as esophageal adenocarcinoma and 5 dying due to other causes.

When comparing ever with never-alcohol consumption for overall survival analysis, there was almost a two-fold increased risk of mortality in unadjusted analysis (HR 1.96 95% CI 1.13–3.38) although this was not statistically significant when adjusted analysis was performed (HR 1.70 95% CI 0.95–3.04). In cancer-specific survival analysis, a similar pattern was observed when comparing ever versus never-alcohol drinkers with a worsened survival in unadjusted analysis (HR 1.99 95% CI 1.12–3.55) and adjusted analysis (HR 1.70 95% CI 0.93–3.11). Mutually adjusting for smoking status did not alter the results shown. These results are presented in Table 3.

**Table 3:** Esophageal adenocarcinoma survival outcomes according to smoking and alcohol status

	Dead *n* = 75	Alive *n* = 55	Unadjusted Hazard ratio (95% CI)	*p*-value	Adjusted Hazard ratio^a^ (95% CI)	*p*-value	Adjusted Hazard ratio^b^ (95% CI)	*p*-value
Overall survival								
Never smoker	21	13	1.00		1.00		1.00	
Current smoker	18	13	1.10 (0.58–2.07)	0.77	1.14 (0.55–2.37)	0.72	1.08 (0.51–2.26)	0.85
Former smoker	26	22	0.93 (0.53–1.67)	0.83	1.25 (0.67–2.35)	0.49	1.10 (0.58–2.10)	0.77
Unknown	10	7	1.33 (0.53–2.41)	0.75	1.19 (0.53–2.66)	0.66	0.38 (0.06–2.32)	0.29
Never alcohol	18	23	1.00		1.00		1.00	
Ever alcohol	45	24	1.96 (1.13–3.38)	0.02	1.70 (0.95–3.04)	0.07	1.67 (0.93–3.01)	0.09
Unknown	12	8	1.64 (0.79–3.42)	0.18	1.73 (0.80–3.80)	0.17	4.29 (0.78–23.68)	0.09
Cancer-specific survival^c^								
Never smoker	19	13	1.00		1.00		1.00	
Current smoker	16	13	1.03 (0.52–2.01)	0.93	1.09 (0.50–2.37)	0.83	1.02 (0.46–2.6)	0.96
Former smoker	26	22	0.96 (0.53–1.75)	0.91	1.35 (0.71–2.58)	0.35	1.19 (0.61–2.31)	0.60
Unknown	9	7	1.04 (0.47–2.29)	0.93	1.14 (0.49–2.64)	0.76	0.39 (0.06–2.47)	0.32
Never alcohol	16	23	1.00		1.00		1.00	
Ever alcohol	43	24	1.99 (1.12–3.55)	0.02	1.70 (0.93–3.11)	0.09	1.66 (0.90–3.06)	0.11
Unknown	11	8	1.57 (0.72–3.39)	0.25	1.66 (0.73–3.75)	0.23	3.98 (0.71–22.37)	0.12

*CI* confidence intervals

^a^Variables included in the adjusted analysis were age at diagnosis, gender, nodal status, circumferential resection margin, lymphovascular invasion, tumor location, and tumor differentiation

^b^Adjustment was made for age at diagnosis, gender, nodal status, circumferential resection margin, lymphovascular invasion, tumor location, tumor differentiation, and smoking in the alcohol analysis and alcohol in the smoking analysis

^c^This analysis included 125 patients as 5 had died due to other causes

Regarding smoking status, there was no apparent difference in overall or cancer-specific survival in either current or former smokers compared to never smokers in both unadjusted and adjusted analysis as shown in Table 3. Additional adjustment for alcohol status did not impact on either set of survival analyses.

### Stratified survival analysis by biomarker expression

The unadjusted association between alcohol consumption and survival in esophageal adenocarcinoma patients according to tumor biomarker expression categories are presented in Table 4. The previously observed increased risk of death for alcohol consumers was more evident in patients within the normal tertile of p53 expression (HR 11.8 95% CI 1.55–89.7), GLUT-1-positive (HR 2.40 95% CI 1.31–4.41), CD 8-positive (HR 2.77 95% CI 1.26–6.09), and HER 2-positive tumors (HR 7.00 95% CI 0.85–57.6), although the latter did not reach statistical significance.

**Table 4:** Esophageal adenocarcinoma survival outcomes according to alcohol status within different biomarker categories

		Never	Ever	Alcohol status				
		Dead/alive	Dead/alive	Never	Ever (overall survival) Unadjusted Hazard ratio (95% CI)	*p*-value	Ever (disease specific survival)^a^ Unadjusted Hazard ratio (95% CI)	*p*-value
p53		*n* = 41	*n*= 69					
240 +	43 (33.1)	10/6	12/7	1.00	1.26 (0.54–2.91)	0.60	1.27 (0.55–2.94)	0.58
80 to < 240	38 (29.2)	1/9	16/6	1.00	12.37 (1.63–93.6)	0.02	11.8 (1.55–89.7)	0.02
< 80	49 (37.7)	7/8	17/11	1.00	1.59 (0.65–3.87)	0.31	1.66 (0.61–4.54)	0.32
HER-2		*n* = 41	*n* = 69					
0	111 (85.4)	17/18	38/21	1.00	1.66 (0.94–2.95)	0.08	1.66 (0.91–3.04)	0.10
1	19 (14.6)	1/5	7/3	1.00	7.00 (0.85–57.6)	0.07	7.00 (0.85–57.6)	0.07
GLUT 1		*n* = 41	*n*= 69					
0	22 (17.1)	3/2	7/6	1.00	0.79 (0.20–3.12)	0.74	1.18 (0.14–10.10)	0.88
1	107 (82.9)	15/21	38/18	1.00	2.5 (1.37–4.57)	0.003	2.40 (1.31–4.41)	0.005
CD 8		*n* = 39	*n* = 65					
0	36 (30)	7/4	15/5	1.00	1.67 (0.68–4.13)	0.26	1.71 (0.65–4.48)	0.27
1	84 (70)	9/19	29/16	1.00	2.78 (1.31–5.89)	0.01	2.77 (1.26–6.09)	0.01

*CI* confidence intervals

^a^This analysis included 125 patients as 5 had died due to other causes

The association between smoking status and survival in esophageal adenocarcinoma patients according to tumor biomarker expression categories is presented in Table 5. No associations between smoking status and survival in esophageal adenocarcinoma were observed according to high or low expression of p53, or positive/negative status for HER2, GLUT-1, or CD8.

**Table 5:** Esophageal adenocarcinoma survival outcomes according to smoking status within different biomarker categories

		Never	Ever	Smoking status				
		Dead/alive	Dead/alive	Never	Ever (Overall survival) Unadjusted hazard ratio (95% CI)	*p*-value	Ever (Disease specific survival)^a^ Unadjusted hazard ratio (95% CI)	*p*-value
p53		*n* = 34	*n* = 79					
240 +	43 (33.1)	6/4	16/9	1.00	1.22 (0.47–3.14)	0.68	1.22 (0.48–3.15)	0.68
80 to < 240	38 (29.2)	8/8	10/11	1.00	0.68 (0.27–1.72)	0.41	0.62 (0.24–1.61)	0.33
< 80	49 (37.7)	7/5	18/15	1.00	1.18 (0.49–2.87)	0.72	1.28 (0.47- 3.47)	0.63
HER-2		*n* = 34	*n* = 79					
0	111 (85.4)	18/12	37/28	1.00	1.07 (0.61–1.88)	0.81	1.07 (0.59–1.93)	0.83
1	19 (14.6)	3/1	7/7	1.00	0.64 (0.16–2.48)	0.52	0.64 (0.16–2.48)	1.00
GLUT 1		*n*= 34	*n*= 79					
0	22 (17.1)	5/3	5/6	1.00	0.75 (0.21–2.64)	0.66	0.54 (0.13–2.18)	0.39
1	107 (82.9)	16/10	39/29	1.00	1.02 (0.57–1.83)	0.95	1.07 (0.59–1.95)	0.83
CD 8		*n* = 30	*n* = 77					
0	36 (30)	5/3	17/6	1.00	1.32 (0.49–3.59)	0.59	1.22 (0.45–3.40)	0.69
1	84 (70)	14/8	26/28	1.00	0.85 (0.44–1.64)	0.63	0.87 (0.44–1.73)	0.88

*CI* confidence intervals

^a^This analysis included 125 patients as 5 had died due to other causes

## Discussion

In this population-representative study, patients with esophageal adenocarcinoma who consumed alcohol had a poorer survival than never drinkers, although statistical significance became attenuated in fully adjusted analyses. In our hypothesis-generating analysis stratified by biomarker expression levels, patients who were ever-alcohol drinkers and had tumors with positive expression of GLUT1, CD8, or were in the middle tertile of p53 expression had significantly poorer survival. Smoking status was not associated with outcomes in patients with esophageal adenocarcinoma, and these non-significant observations remained in the analyses stratified by selected biomarker expression levels.

To our knowledge, only three studies and one meta-analysis have previously investigated the association between alcohol consumption and survival in esophageal adenocarcinoma [10, 12–14]. A contributing factor to this lack of research may be the short life expectancy associated with this disease, meaning it is difficult to study epidemiological factors in relation to survival. Although none of the adjusted results in these studies reached statistical significance, some did demonstrate a non-significant poorer survival in esophageal adenocarcinoma patients who were alcohol drinkers in line with the current study findings [10, 12–14]. It should be noted that previous studies included both curative and palliative patients, whereas the current study only included patients undergoing treatment with a curative intent. Therefore, by focusing on the patients with the most favorable prognosis, we maximized the opportunity to see any effect of these lifestyle factors on survival. For example, in a study reported by Thrift et al., 38% of patients were palliative and a difference in survival outcomes between lifestyle groups may be more difficult to identify [12].

The largest study to date was performed in Australia and included 362 patients with esophageal adenocarcinoma [12]. Alcohol consumption was assessed by providing questionnaires which assessed patient consumption between the ages of 0–29, 30–49, and > 50 which was then used to divide patients into groups of average lifetime alcohol consumption of < 1 drink, 1–6, 7–20 ,and greater than 20 drinks per week with one drink considered to be equivalent to 10 g of alcohol. In adjusted analysis, when comparing outcomes in the groups who consumed alcohol regularly to the group who consumed less than 1 drink per week, there was no significant difference in outcomes, although in the group who drank 7–20 drinks per week, there was a trend of worse survival (HR 1.52 95% CI 0.98–2.37) [12].

The second largest study was carried out by Trivers et al. who performed a multicentered population-based case–control study in the USA with 293 cases of esophageal adenocarcinoma of various stages. In their unadjusted analysis, there was no significant difference in survival between never drinkers and ever drinkers (HR 1.08 95% CI 0.81–1.44) [10]. The third study, a nationwide case–control study performed by Sundelof et al. in Sweden [13] included 177 patients with esophageal adenocarcinoma of which 102 underwent esophagectomy and 75 did not. Alcohol intake was based on consumption 20 years prior to the questionnaire and was divided into never, 1–15 g per week, 16–70 g per week, and more than 70 g per week. There was no difference in survival in any of the alcohol drinker groups compared to the never group [13].

The studies by Thrift et al. and Sundelof et al. were able to be combined in a previous meta-analysis, and although results did not reach a level of statistical significance, they suggested that survival may be worse for moderate drinkers compared to never drinkers (HR 1.34 95% CI 0.95–1.89) [14]. However, there was a lack of dose–response association observed, since weaker survival estimates were reported when heavy alcohol consumption was compared to never consumption (HR 1.01 95% CI 0.70–1.47) [14]. Unfortunately, we were unable to assess dose–response associations for alcohol and smoking, due to the lack of reporting this level of detail in the hospital case notes, which were retrospectively reviewed. Although alcohol is not associated with the risk of esophageal adenocarcinoma [9], these results suggest that there is an association with survival and highlights the necessity of larger studies to investigate these findings further in order to inform potential adjuvant lifestyle interventions.

A limited number of studies have investigated the association between tobacco smoking and survival in patients with esophageal adenocarcinoma, and their results are in agreement with our findings [10–13]. In a meta-analysis by McMenamin et al., these studies were combined and there was no significant difference in survival outcomes when comparing current (HR 0.99 95% CI 0.73–1.36) or former (HR 0.88 95% CI 0.68–1.14) smokers with never smokers, respectively [14]. Our hypothesis-generating results exploring the association between tobacco smoking and survival in esophageal adenocarcinoma patients according to the expression of selected biomarkers also did not reveal significant associations.

The hypothesis-generating results within the alcohol status categories were more interesting and highlight the necessity of further research in this area. To date, there has only been one study (performed on the same cohort of patients as this study) which has investigated the role of GLUT1 as a biomarker in esophageal adenocarcinoma, and positive expression was associated with a poorer prognosis [17]. This is, however, the first study to identify a potential interaction between alcohol, GLUT1 expression, and survival in esophageal adenocarcinoma. The poorer outcomes in ever drinkers with CD8 positive, or normal p53, tumor expression indicate that further mechanistic studies are warranted to verify the biological plausibility of a potential underlying interaction with alcohol intake in relation to prognosis.

This study has several strengths. It adds to the small pool of studies that have been performed in this area and compliments their findings. Furthermore, it is the first study to apply molecular epidemiology pathology methods to study the interaction between lifestyle factors, biomarker expression, and survival. This study has a number of limitations. Firstly, the collected data did not include details on patient comorbidities and therefore adjustments were not made for these in the survival analysis. Secondly, this study has a relatively small sample size, particularly for stratified analyses by biomarker expression, although our cohort size of 130 patients is typical of this relatively rare disease site. Nevertheless, our study provides support for future larger studies to be conducted. Thirdly, all patients in this study had surgically resectable disease, meaning this cohort represents patients with more favorable prognosis, and we cannot deduce if smoking or alcohol consumption impacts upon the outcome in patients with more advanced disease.

In conclusion, this study demonstrates that ever-alcohol consumption may have a negative impact on survival in patients with esophageal adenocarcinoma and in particular those patients with CD8- or GLUT1-positive tumors, or with expression of p53 within the middle tertile. However, cigarette smoking was not shown to be associated with survival in patients with esophageal adenocarcinoma. Larger studies are needed to confirm these findings as lifestyle advice could potentially be promoted not just on the basis of decreasing the risk of developing cancer or other medical diagnoses but also for improving outcomes should a patient receive an esophageal adenocarcinoma diagnosis. Studies are also required to investigate the impact of lifestyle change such as cigarette smoking and alcohol cessation at the time of a cancer diagnosis as lifestyle advice could form patients part of a patient’s treatment strategy.
